# Vaccination Against HMGB1 Protects Mice from CCl_4_-Induced Liver Injury, Inflammation, and Fibrosis

**DOI:** 10.1007/s10753-026-02578-4

**Published:** 2026-08-01

**Authors:** Qian Li, Hanchao Li, Yongwei Huo, Hong Tian, Li Zhu, Xiaoyan Zheng, Wengang Sun, Xueting Zhang, Zhiming Hao

**Affiliations:** 1 Department of Rheumatology, The First Affiliated Hospital of Xi’an Jiaotong University, 277 West Yanta Road, Xi’an, Shaanxi 710061 China; 2 Center of Reproductive Medicine, School of Basic Medical Sciences, Xi’an Jiaotong University Health Science Center, Xi’an, China; 3 Department of Rehabilitation Medicine, Shaanxi Provincial People’s Hospital, Xi’an, China

**Keywords:** High mobility group box 1 (HMGB1), Liver fibrosis, Receptor for advanced glycation end products (RAGE), Toll-like receptor 4 (TLR4), Vaccine

## Abstract

Extracellular high mobility group box 1 (HMGB1) serves as a key damage-associated molecular pattern that drives the pathological process of inflammatory liver injury; however, its role in liver fibrosis and the underlying mechanisms need to be elucidated. To investigate the proinflammatory and profibrotic roles of extracellular HMGB1, we used an adenovirus-mediated system to measure HMGB1 secretion in mice. We subsequently constructed two recombinant proteins, PfTrx-HMGB1^17^(95 − 111) and HBcΔ-HMGB1^34^(150 − 183), targeting the interactions of HMGB1 with its receptors, namely, toll-like receptor 4 (TLR4) and receptor for advanced glycation end products (RAGE), respectively. Mice were immunized with the two recombinant proteins to assess neutralizing antibody induction and the protective effects of the HMGB1 vaccines against carbon tetrachloride (CCl_4_)-induced liver fibrosis. Our results revealed the nucleocytoplasmic translocation of HMGB1 in the livers of both humans and mice with fibrosis. Extracellular HMGB1 induced liver inflammation and fibrogenesis in mice. Immunization with the two HMGB1 vaccines resulted in the production of high levels of anti-HMGB1 neutralizing antibodies, which inhibited the activation of hepatic stellate cells (LX-2) and the production of proinflammatory and profibrotic factors in RAW 264.7 cells. Furthermore, vaccination with these compounds significantly protected against CCl_4_-induced liver injury, inflammation, and fibrosis in mice. These findings demonstrate that extracellular HMGB1, by binding to TLR4 or RAGE, is pivotal in driving liver inflammation and fibrosis. Vaccination against HMGB1 through disruption of interaction with TLR4 or RAGE represents a promising therapeutic strategy for hepatic fibrosis.

**Supplementary Information:**

The online version contains supplementary material available at https://doi.org/10.1007/s10753-026-02578-4.

## Introduction

High mobility group box 1 (HMGB1), a nonhistone chromosomal protein, is widely expressed in mammalian cells, and its functions vary depending on its subcellular localization. Nuclear HMGB1 acts as a DNA chaperone that maintains nucleosome integrity and participates in DNA repair and gene transcription [1]. When released into the extracellular space, HMGB1 functions as a prototypical damage-associated molecular pattern by binding to specific cell-surface receptors—such as the receptor for advanced glycation end products (RAGE) and Toll-like receptors (TLRs) 2, 4, and 9—thereby triggering inflammation and adaptive immune responses [1, 2].

Hepatic fibrosis results from persistent liver injury and inflammation and ultimately progresses to cirrhosis, which is characterized by portal hypertension, liver failure, and an increased risk of hepatocellular carcinoma [3, 4]. Recent studies have reported the involvement of HMGB1 in the pathogenesis of acute liver injury, inflammation, and liver fibrosis [5–21]. Serum and hepatic HMGB1 levels are elevated in patients with chronic hepatitis C virus infection, primary biliary cholangitis, and ‌alcohol-associated steatohepatitis (ASH)‌, as well as in mice with liver fibrosis induced by carbon tetrachloride (CCl_4_), thioacetamide, cholestasis (bile duct ligation, BDL), ASH, or nonalcoholic steatohepatitis (NASH) induced by a methionine- and choline-deficient diet [6]. Administration of a low dose of exogenous recombinant human HMGB1 (rhHMGB1) exacerbates CCl_4_-induced liver injury [6, 7]and fibrosis [6] in mice. In vitro studies have shown that HMGB1 promotes the proliferation [8, 9], migration [6], and activation of hepatic stellate cells (HSCs) via TLR4- or RAGE-mediated pathways [7, 9]. It also promotes autophagy in HSCs by activating the extracellular signal-regulated kinase/c-Jun N-terminal kinase (JNK) mitogen-activated protein kinase pathway while suppressing the mammalian target of rapamycin/STAT3 signaling pathways [10]. HMGB1-neutralizing antibodies alleviate acetaminophen-induced acute liver injury [11]. Knockdown of HMGB1 using small interfering RNA (siRNA) [12] or short hairpin RNA (shRNA) [13], genetic ablation of HMGB1 in hepatocytes [6, 14] or myeloid cells [6], and treatment with peptide-based antagonists (HMGB1 A-box [15] or soluble RAGE [6]) protect mice from CCl_4_-induced liver fibrosis. The HMGB1 A-box also attenuates fibrosis in melanocortin-4 receptor-deficient NASH models [16]. Additionally, inhibition of the HMGB1 pathway contributes to the antifibrotic effects of several compounds, including curcumin [17], nilotinib [18], glycyrrhizin [19], and ethyl pyruvate [20], on murine liver fibrosis. Despite this supporting evidence, direct proof of an in vivo profibrotic role for HMGB1 remains elusive. HMGB1 has a complex structure consisting of three domains: an A-box (amino acids [aa] 7–79), a B-box (aa 89–162), and a C-tail (aa 186–215). Extracellularly, full-length HMGB1 and the B-box exhibit proinflammatory effects, whereas the A-box counteracts the proinflammatory activity of the B-box [1]. Additionally, extracellular HMGB1 exerts distinct biological effects depending on its binding to specific receptors. Therefore, simply reducing HMGB1 expression, inhibiting its release, or generating antibodies against full-length HMGB1 represents an overly simplistic therapeutic approach. The development of safe and effective domain-specific inhibitors that disrupt the interactions between HMGB1 and its receptor is a key focus of ongoing research [21].

In our previous study, we prepared vaccines by coupling HMGB1 peptide segments with keyhole limpet hemocyanin (KLH) and evaluated their therapeutic effects on CCl_4_-induced mouse hepatic fibrosis. The results demonstrated that immunization with antigenic peptides located in the A-box (HMGB1 aa 53–63, aa 67–78) not only failed to confer protection but also significantly exacerbated fibrosis. In contrast, the vaccine targeting the epitope aa 95–111 showed significant protective effects against CCl_4_-induced hepatic fibrosis in moice, suggesting that vaccines targeting the HMGB1 B-box could effectively inhibit liver fibrosis. Based on these results, the present study first examined the expression of HMGB1 in fibrotic liver tissues. We then evaluated the proinflammatory and profibrotic effects of extracellular HMGB1 using an adenovirus-mediated system for HMGB1 secretion in mice. We subsequently constructed two recombinant HMGB1-based vaccines designed to target the interactions between HMGB1 and either TLR4 or RAGE and assessed their ability to elicit neutralizing anti-HMGB1 antibodies. Finally, we evaluated the protective effects of these vaccinations against CCl_4_-induced hepatic injury, inflammation, and fibrosis in mice.

## Materials and Methods

### Human and Mouse Liver Tissues

Normal liver tissue samples were collected from patients who underwent surgical resection for metastatic hepatic malignancies. Cirrhotic liver samples were obtained from patients who underwent liver transplantation at the First Affiliated Hospital, Xi’an Jiaotong University. Informed consent was obtained from each patient after surgery, and the study was approved by the Institutional Review Board. Liver tissues of mice with fibrosis induced by CCl_4_ and BDL were obtained from our previous studies [22, 23].

### Animals

Specific pathogen-free, 6-week-old male ICR mice were purchased from the Experimental Animal Center, School of Basic Medical Sciences, Xi’an Jiaotong University Health Science Center. The mice were housed in the animal facilities of our laboratory, kept at 25 °C with a 12-h light/dark cycle, allowed free access to a chow diet and water, and were acclimatized for one week prior to the experiments. The maintenance of the mice and all the experimental protocols were approved by the Institutional Animal Ethics Committee of Xi’an Jiaotong University.

### Generation of Recombinant Adenoviruses

To validate the proinflammatory and profibrotic effects of extracellular HMGB1 in mouse livers, we constructed an adenoviral vector carrying human HMGB1 cDNA fused to the murine immunoglobulin kappa signal peptide (MIKSP) sequence at its 5′ terminus to enable HMGB1 secretory expression (AdMIKSP-HMGB1). In addition, an adenoviral vector carrying full-length human HMGB1 cDNA (AdHMGB1) was generated to induce HMGB1 overexpression. An adenovirus expressing green fluorescent protein (GFP) (AdGFP) served as a control.

Replication-incompetent adenoviruses (type 5, ∆E1/E3) AdHMGB1, AdMIKSP-HMGB1, and AdGFP were generated using the AdEasy™ Adenovirus Vector System (Stratagene, La Jolla, CA). To construct an adenovirus vector carrying the *HMGB1* gene, the coding region of human *HMGB1* was amplified from a plasmid harboring full-length human *HMGB1* cDNA (Sino Biological, Inc., Beijing, China) via PCR using the following primers: 5′-GG*TACCAT*GGGCAAAGGAGATCCTAAG-3′ (forward, KpnI site underlined) and 5′-CCC*CTCGAG*TTATTCATCATCATCATCTTC-3′ (reverse, XhoI site underlined). The coding region of MIKSP was fused to the N-terminus of HMGB1. *MIKSP* was obtained by overlap extension PCR using the primers: 5′-GG*GGTACC*ATGGAGACAGACACACTCCTGCTATGGGTACTGCTGCTGTGGGTTCCAG-3′ (forward, KpnI site underlined) and 5′-G*GAATTC*CGGCTGGGCCGCGTCACCAGTGGAACCTGGAACCCACAGCAGCAGTAC-3′ (reverse, EcoRI site underlined). The coding region of human *HMGB1* was amplified using a forward primer (5′-CC*GAATTC*ATGGGCAAAGGAGATCCTAAG-3′, EcoRI site underlined) and the reverse primer listed above. PCR products were digested and subcloned into pShuttle-CMV. Linearized pShuttle-CMV-HMGB1 or pShuttle-CMV-MIKSP-HMGB1, together with pAd-easy-1, were transformed into *E. coli* BJ5183 for homologous recombination. Packaging and amplification of the recombinant adenoviruses were carried out in HEK 293T cells. Viral titers were determined using plaque-forming assays and are expressed as plaque-forming units (PFUs).

### Adenovirus-Mediated HMGB1 Secretion in Mice in Liver Inflammation and Fibrosis

Forty-eight mice were randomly divided into four groups: the NC, AdGFP, AdHMGB1, and AdMIKSP-HMGB1 groups. The adenoviruses were intraperitoneally (i.p.) administered at a dose of 1 × 10^10^ PFUs per mouse in the three adenoviral groups, whereas mice in the NC group received an equal volume of phosphate-buffered saline (PBS) instead. Six mice in each group were euthanized after one week to detect liver inflammation. To evaluate the profibrotic effects of secretory HMGB1, the remaining 24 mice were treated with the adenoviruses as described above, except that two doses of adenoviruses were administered two weeks apart, and the mice were euthanized two weeks after the second injection.

### Preparation of the Recombinant Proteins

The plasmid pET26b-PfTrx, containing the coding region of *PfTrx*, was a gift from Dr. Simone Ottonello at the Department of Life Sciences, Biochemistry and Molecular Biology Unit, University of Parma, Italy [24]. The coding region of *HMGB1* aa 95–111 (KRPPSAFFLFCSEYRPK, HMGB1^17^(95–111)) was amplified using the primers 5′-*CTGCAG*GGCCCAAGAGGCCTCCTTCG-3′ (forward, PstI site underlined) and 5′-*GGATCC*TGGGCGATACTCAGAGCAG-3′ (reverse, BamHI site underlined) and inserted into PfTrx between the PstI and BamHI sites, yielding pET26-PfTrx-HMGB1^17^(95–111).

HMGB1^34^(150–183) was constructed by inserting the coding region corresponding to HMGB1 aa 150–183 (KLKEKYEKDIAAYRAKGKPDAAKKGVVKAEKSKK) into HBcΔ. The coding region was amplified using the following primers: 5′-*CTCGAG*AAGCTGAAGGAAAAATACGAAAAGG-3′ (forward, XhoI site underlined) and 5′-*GGATCC*TTTCTTGCTTTTTTCAGCCTTGAC-3′ (reverse, BamHI site underlined). The PCR product was inserted into pGEMEX-1-HBcΔ, yielding the recombinant plasmid pGEMEX-1-HBcΔ-HMGB1^34^(150–183). It was then digested with EcoRI and HindIII and subsequently inserted into pET-28a(+), yielding pET28-HBcΔ-HMGB1^34^(150–183).

pET26-PfTrx, pET26-PfTrx-HMGB1^17^(95–111), pET28-HBcΔ, and pET28-HBcΔ-HMGB1^34^(150–183) were transformed into *E. coli* BL21(DE3). Protein expression and purification were monitored via sodium dodecyl sulfate‒polyacrylamide gel electrophoresis (SDS‒PAGE) followed by Coomassie Brilliant Blue staining. Correct folding of PfTrx was verified using insulin reduction assays. Purification of HBcΔ and HBcΔ-HMGB1^34^(150–183) was performed via size-exclusion chromatography on an ÄKTAPrime™ Plus purification system (GE Healthcare, Fairfield, CT), and the formation of virus-like particles (VLPs) was verified using electron microscopy (H600 transmission electron microscope, Hitachi).

Endotoxin/LPS removal to levels less than 2 EU/mL was confirmed for each sample using the QCL-1000™ Endpoint Chromogenic LAL assay (Lonza). The purified proteins were dialyzed against PBS, and glycerol was added to a final concentration of 10% (v/v). The proteins were filtered through 0.22 μm sterilizing filters, aliquoted, and stored at − 20 °C.

### Verification of the Antigenicity of the Recombinant Proteins

Thirty mice were randomly assigned to five groups—PBS, PfTrx, PfTrx-HMGB1^17^(95–111), HBcΔ, and HBcΔ-HMGB1^34^(150–183)—and received i.p. immunizations of the recombinant proteins (50 µg) or an equal volume of PBS biweekly for four doses. The recombinant proteins were emulsified in Freund’s complete adjuvant (Sigma–Aldrich, St. Louis, MO, USA) for the first immunization and in Freund’s incomplete adjuvant (Sigma–Aldrich) for the subsequent immunizations. Antibody production was monitored using enzyme-linked immunosorbent assay (ELISA). One week after the fourth immunization, three mice per group were euthanized, and blood was collected to isolate serum. Mouse serum IgG was purified using octanoic acid–ammonium sulfate precipitation. Antibody levels in the remaining mice were monitored biweekly until euthanasia, 24 weeks after the first immunization. To evaluate vaccine safety, the liver, lungs, heart, and kidneys were harvested, fixed in 10% formalin, sectioned, and stained with hematoxylin and eosin to assess any adverse effects.

### Cecal Ligation and Puncture (CLP) Model

A total of 50 mice were equally assigned to the PBS, PfTrx, PfTrx-HMGB1^17^(95–111), HBcΔ, and HBcΔ-HMGB1^34^(150–183) groups and immunized as described above. One week after the fourth immunization, CLP was performed as previously described [25]. Briefly, following 3% isoflurane anesthesia, the cecum was ligated 1 cm from the distal end and through-and-through punctured with a 19-gauge needle to allow fecal extrusion. The abdomen was then closed, and the mice were resuscitated with 1 mL of prewarmed 0.9% saline subcutaneously. No antibiotics were administered.

### ELISA

Mouse sera were serially diluted and added to plates precoated with PfTrx, HBcΔ, or rhHMGB1 (100 ng/well, Sigma-Aldrich). The plates were then incubated at 37 °C for 1 h. After the samples were washed with PBST, goat anti-mouse IgG-horseradish peroxidase (HRP) (1:10,000 in 10% fetal bovine serum [FBS]/PBST) was added, and the samples were incubated for another hour. The plates were subsequently washed five times with PBST, after which the chromogenic substrate solutionwas added. The absorbance was measured at 450 nm (OD_450_).

For competitive ELISA, rhHMGB1-HRP (1:10,000 in 2% human serum albumin/PBST) was preincubated with serially diluted purified anti-PfTrx-HMGB1^17^(95–111) (0.00, 0.04, 0.20, 1.00, and 5.00 µg/mL) or anti-HBcΔ-HMGB1^34^(150–183) (diluted at 0, 1, 10, 100, and 1000 ng/mL, respectively) antibodies at 37 °C for 1 h. In parallel, anti-PfTrx (5.00 µg/mL), anti-HBcΔ (1000 ng/mL), and NC (1000 ng/mL) antibodies served as controls. The mixtures were then added to plates coated with recombinant TLR4 (50 ng/well; Sino Biological) or RAGE (25 ng/well; Sino Biological). The plates were incubated at 37 °C for another hour and then washed five times with PBST, after which the optical density (OD) was measured at 450 nm (OD_450_). The inhibition ratio (%) was calculated as follows: 100 × ([OD_450_ value of control antibody] – [OD_450_ of anti-HMGB1 antibody])/(OD_450_ value of the control antibody). A competitive ELISA was performed in six independent experiments. For each independent experiment, samples were measured in duplicate wells.

### CCl_4_-Induced Hepatic Fibrosis

Sixty mice were randomly assigned to six groups: the NC, CCl_4_, PfTrx/CCl_4_, PfTrx-HMGB1^17^(95–111)/CCl_4_, HBcΔ/CCl_4_, and HBcΔ-HMGB1^34^(150–183)/CCl_4_ groups. Mice in the PfTrx/CCl_4_, PfTrx-HMGB1^17^(95–111)/CCl_4_, HBcΔ/CCl_4_, and HBcΔ-HMGB1^34^(150–183)/CCl_4_ groups were immunized as described above. One week after the fourth immunization, the mice in the five CCl_4_ groups received i.p. injections of CCl_4_ (1 mL/kg dissolved in olive oil to a final concentration of 20%), twice weekly for six weeks to induce liver fibrosis [22]. The NC group received an equal volume of olive oil instead. The mice were euthanized 12 h after the final CCl_4_ injection, and serum and liver tissues were collected.

### Aminotransferase, Tissue Myeloperoxidase (MPO) and Superoxide Dismutase (SOD) Activities and Malondialdehyde (MDA) Content

Serum alanine aminotransferase (ALT), aspartate aminotransferase (AST), lactate dehydrogenase (LDH), liver myeloperoxidase (MPO), malondialdehyde (MDA), and superoxide dismutase (SOD) levels were determined with commercially available kits (Nanjing Jiancheng Bioengineering Institute, Nanjing, China).

### Histology and Immunohistochemistry (IHC)

Formalin-fixed, paraffin-embedded liver tissues were cut into 5-µm-thick sections, deparaffinized, rehydrated, and subsequently stained with hematoxylin and eosin and Sirius Red. Immunohistochemistry was performed using a Histostain^TM^-Plus SP Kit (Thermo Fisher Scientific, Carlsbad, CA, USA) with mouse anti-alpha smooth muscle actin (α-SMA, Thermo Fisher Scientific, CAPA5-85070, 1:800), rabbit anti-HMGB1 (ProteinTech, Wuhan, China, 10829-1-AP, 1:500), rabbit anti-Col1A2 (ProteinTech, 34204-1-AP, 1:500), rabbit anti-Col3A1 (ProteinTech, 22734-1-AP, 1:500), rabbit anti-vimentin (ProteinTech, 10366-1-AP, 1:2000), and mouse anti-cleaved caspase 3/P17/P19 (ProteinTech, 68773-1-Ig, 1:1000) antibodies as primary antibodies.

### Terminal Deoxynucleotidyl Transferase dUTP Nick End Labeling (TUNEL) Assay

Apoptotic hepatocytes were detected in situ using a TUNEL peroxidase detection kit (DeadEnd™ Colorimetric TUNEL System; Promega, Madison, WI) according to the manufacturer’s instructions. The nuclei were counterstained with hematoxylin. Apoptosis was quantified by counting the number of TUNEL-positive nuclei in at least 10 randomly selected high-power fields (400× magnification). The results are expressed as the apoptotic index (AI).

### Quantitative Real-Time Polymerase Chain Reaction (qRT − PCR)

Total RNA was extracted using TRIzol reagent (Thermo Fisher Scientific, Carlsbad, CA, USA) and quantified with a NanoDrop 2000 spectrophotometer (Thermo Fisher Scientific, Carlsbad, CA). Reverse transcription was performed using a PrimeScript™ RT Reagent Kit (TaKaRa, Dalian, China). The relative mRNA abundance in each sample was determined via qRT‒PCR using gene-specific primers (Supplementary Table 1) and SYBR^®^ Premix ExTaq™ II (TaKaRa, Dalian, China) on an iQ™ Multicolor Real-Time PCR Detection System (Bio-Rad, Hercules, CA). The data were analyzed using the 2^−ΔΔCT^ method, with β-actin used as the internal control.

### Western Blotting

For routine western blotting analysis, total cell and tissue lysates were prepared. To specifically evaluate HMGB1 translocation, nuclear and cytoplasmic proteins from mouse liver tissues were extracted using a Nuclear and Cytoplasmic Protein Extraction Kit (KeyGen Biotech, Nanjing, China) according to the manufacturer’s instructions. Equal amounts of proteins (100 µg) were subjected to SDS‒PAGE and transferred onto nitrocellulose membranes. After blocking for 1 h in 10% skim milk, the membranes were incubated overnight with primary antibodies at 4 °C. Mouse anti-α-SMA (1:1,000), rabbit anti-HMGB1 (1:1,000), rabbit anti-vimentin (1:5,000), rabbit anti-GFP (ProteinTech, 1:5,000), mouse anti-GAPDH (ProteinTech, 60004-1-Ig, 1:50,000), and rabbit anti-lamin B1 (ProteinTech, 12987-1-AP, 1:50,000) were used as primary antibodies. HRP-conjugated goat anti-mouse IgG (ProteinTech, SA00001-1, 1:10,000) or HRP-conjugated goat anti-rabbit IgG (ProteinTech, SA00001-2, 1:10,000) were used as secondary antibodies. Finally, the blots were developed using an enhanced chemiluminescence reagent (Applygen Technologies Inc., Beijing, China). The levels of target proteins in total lysates were normalized to those of GAPDH. For the nuclear and cytoplasmic fractions, HMGB1 levels were normalized to those of GAPDH (for cytoplasmic proteins) or lamin B1 (for nuclear proteins).

### Cell Culture and Experiments

HeLa cells, the human monocytic leukemia cell line THP-1, the human HSC cell line LX-2 and the mouse macrophage line RAW 264.7 were obtained from the Type Culture Collection of the Chinese Academy of Sciences (Shanghai, China). All the cells were maintained in Dulbecco’s modified Eagle’s medium (DMEM) supplemented with 10% FBS (Gibco/BRL, Melbourne, Australia) at 37 °C in a humidified atmosphere containing 5% CO_2_. For the experiments, the cells were seeded in 6-well plates at a density of 1 × 10^6^ cells/well. All in vitro experiments were performed in three independent experiments. For each independent experiment, cells were seeded in triplicate wells for each treatment group.

To evaluate the neutralizing effects of antibodies elicited by vaccination, THP-1, LX-2 and RAW 264.7 cells in 6-well plates were divided into six groups: blank control, rhHMGB1, rhHMGB1 + anti-PfTrx antibodies, rhHMGB1 + anti-PfTrx-HMGB1^17^(95–111) antibodies, rhHMGB1 + anti-HBcΔ antibodies and rhHMGB1 + anti-HBcΔ-HMGB1^34^(150–183) antibodies. Cells were cultured in complete DMEM containing polymyxin B (70 U/mL). rhHMGB1 (100 ng/mL) and purified mouse IgGs at various concentrations were added to the medium. Because rhHMGB1 may contain both reduced and disulfide forms, which potentially differ in their higher-order structures and bioactivities, we also tested the neutralizing effects of the antibodies against disulfide HMGB1 (HMGBiotech, Milano, Italy) in LX-2 and RAW 264.7 cells. THP-1 cells were harvested 4 h later for RNA extraction, while LX-2 and RAW 264.7 cells were collected 24 h later for total RNA or protein extraction.

### Statistical Analysis

Quantitative data are presented as the mean ± standard error of the mean (SEM). Statistical analyses were performed using GraphPad Prism software version 10.4.2 (GraphPad Software Inc., La Jolla, CA, USA). Statistical significance among multiple groups was assessed using one-way analysis of variance (ANOVA), followed by Tukey’s post hoc test for pairwise comparisons. Differences between two groups were analyzed using an unpaired Student’s t test. A *P* value of < 0.05 was considered to indicate statistical significance.

## Results

### Upregulation of HMGB1 Expression is Accompanied by Nucleo-Cytoplasmic Translocation in Fibrotic Livers

**Fig. 1:**
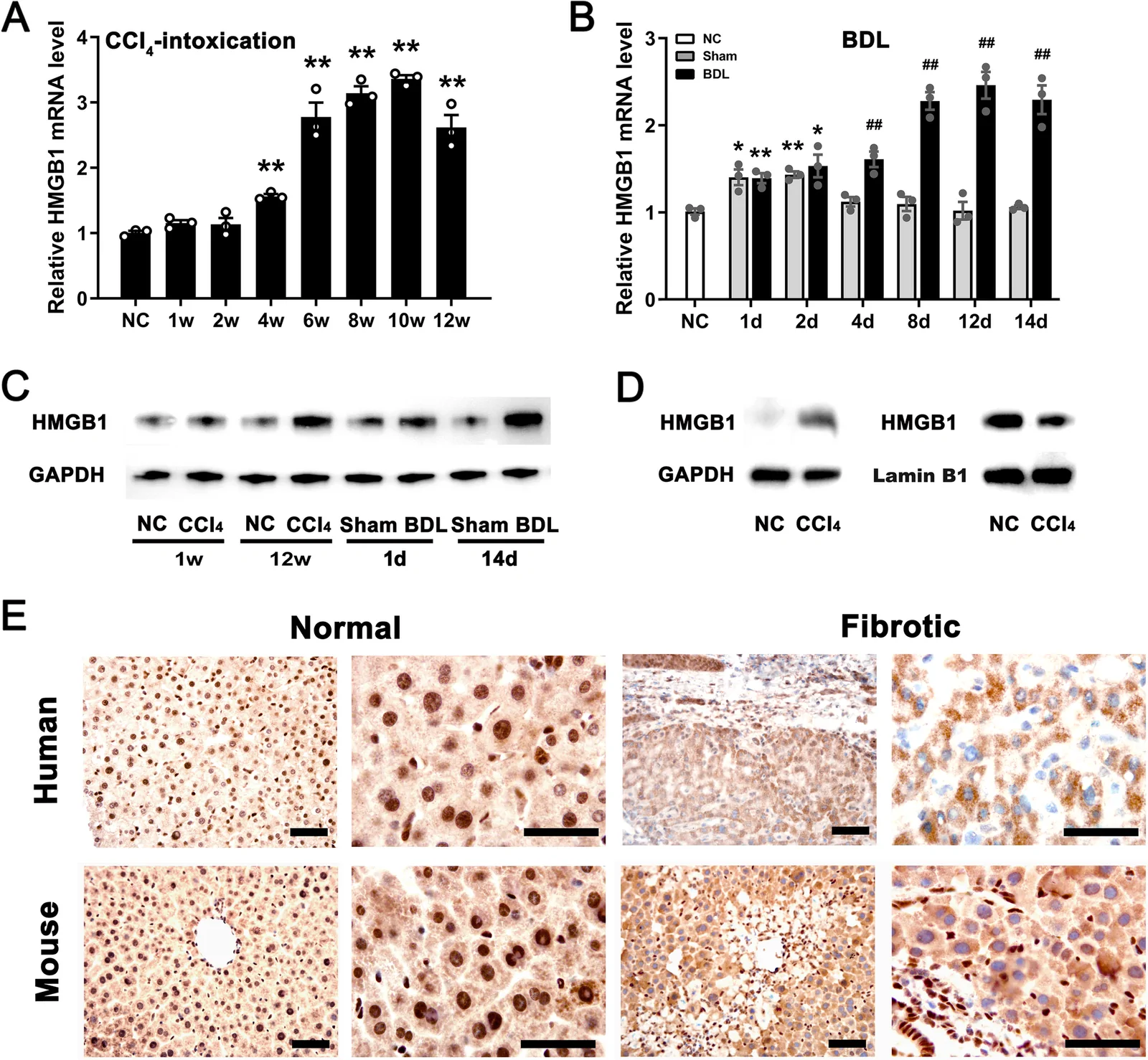
Expression and subcellular localization of HMGB1 in fibrotic livers. **A**–**B** qRT‒PCR determination of HMGB mRNA in CCl_4_- and BDL-induced mouse fibrotic liver tissues. *n* = 3 mice per group at each time point. ^*^P < 0.05 and ^**^P < 0.01 vs. NC group. ^##^*P* < 0.01 vs. Sham group at the same time point. **C**–**D** Western blotting analysis of total HMGB1, as well as cytoplasmic and nuclear HMGB1 expression in fibrotic mouse livers. **E** IHC for HMGB1 in both human (upper) and mouse (lower, CCl_4_-induced) fibrotic liver tissues. Scale bars = 50 μm. Data are shown as mean ± SEM

We first analyzed the expression profiles and subcellular localization of HMGB1 in both mouse and human fibrotic liver tissues. In CCl_4_-treated mice, hepatic HMGB1 mRNA expression began to increase two weeks after treatment, peaked at week 10, and then gradually decreased (Fig. 1A). In BDL mice, hepatic HMGB1 mRNA levels slightly increased in both the sham and BDL groups within 2 d after surgery, likely due to the surgical injury. Subsequently, the HMGB1 mRNA level decreased in the sham group but increased in the BDL group, remaining elevated until the end of the experiment (day 14) (Fig. 1B). The upregulated expression of HMGB1 in fibrotic mouse liver tissues was further confirmed by western blotting analysis (Fig. 1C). Nuclear and cytoplasmic fractionation revealed that HMGB1 was predominantly localized in the nuclei of normal livers, whereas a strong HMGB1 signal was observed in the cytoplasm of fibrotic mouse livers (Fig. 1D). Consistent with these findings, IHC revealed that HMGB1 was primarily nuclear in normal hepatocytes, but exhibited strong cytoplasmic staining in fibrotic human and mouse (CCl_4_-induced) livers (Fig. 1E). These results align with previous findings [6], demonstrating that HMGB1 upregulation during liver fibrosis is accompanied by its nucleo-cytoplasmic translocation. This translocation is an essential prerequisite for its extracellular release, after which secreted HMGB1 binds to receptors such as TLR4 and RAGE to orchestrate proinflammatory and profibrotic cascades that accelerate liver fibrogenesis.

### Adenovirus-Mediated HMGB1 Secretion Induces Inflammation and Fibrogenesis in Mouse Livers

**Fig. 2:**
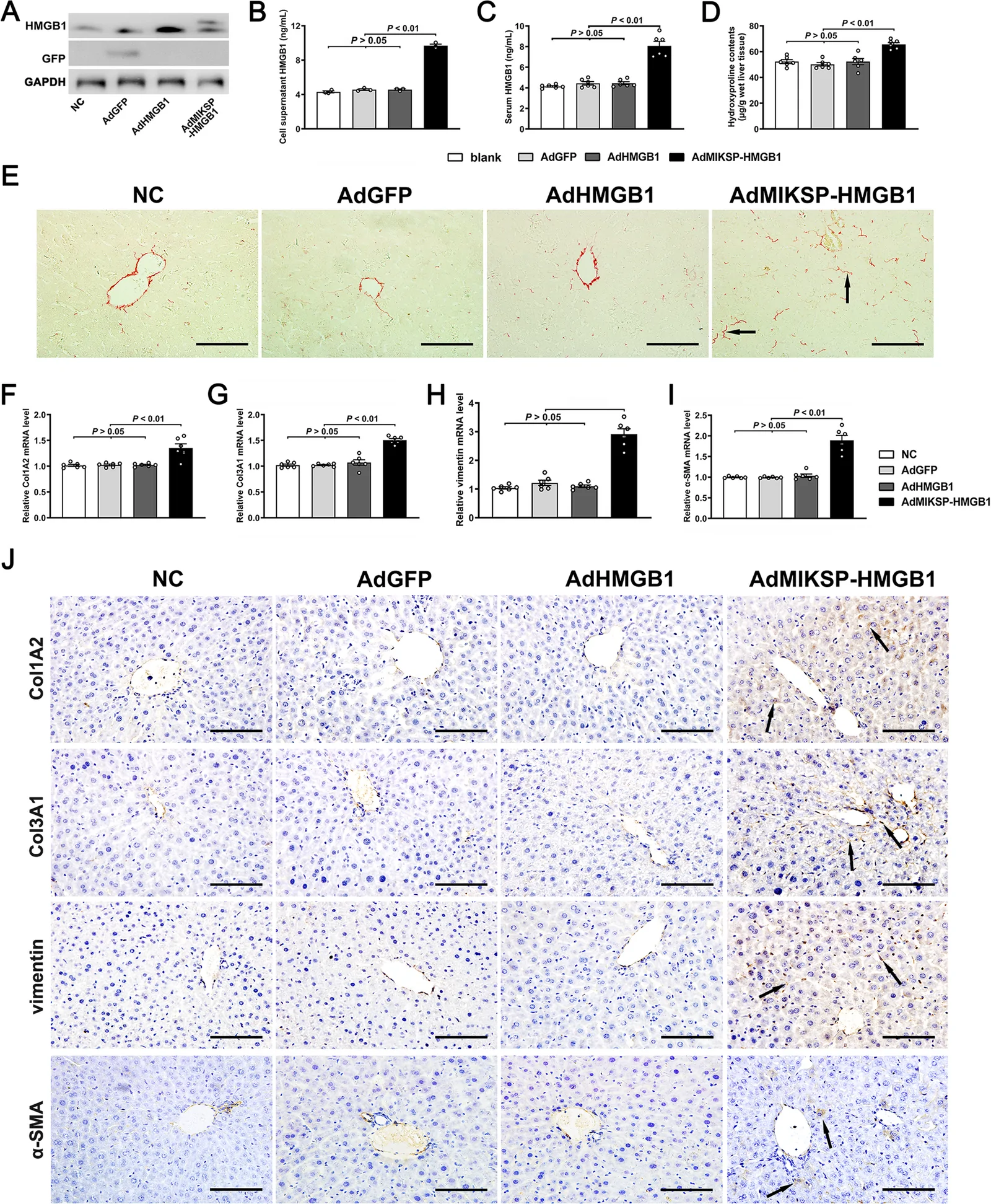
Adenovirus-mediated HMGB1 secretion induces hepatic fibrogenesis in mice. **A** Western blotting analysis of HMGB1 and GFP in adenovirus-infected HeLa cells. **B** ELISA for HMGB1 concentrations in the culture media. *n* = 3 independent experiments. **C** ELISA for mouse serum HMGB1 concentrations one week after the infection. *n* = 6 mice per group. **D**–**E** Hepatic hydroxyproline contents and Sirius Red staining of the mouse liver tissues two weeks after the second adenovirus-infection. *n* = 6 mice per group. Scale bars = 100 μm. **F**–**I** Col1A2, Col3A1, vimentin and α-SMA mRNA expression in the mouse livers. *n* = 6 mice per group. **J** IHC for Col1A2, Col3A1, vimentin, and α-SMA in mouse liver tissues. Scale bars = 100 μm. Data are shown as mean ± SEM.

To verify that MIKSP mediated HMGB1 secretion, HeLa cells were infected with the adenoviruses (multiplicity of infection [MOI] = 100). After 48 h, the complete medium was replaced with serum-free medium, and after an additional 24 h, both the cells and the medium were collected for western blotting and ELISA analyses, respectively. Western blotting revealed that AdHMGB1-infected HeLa cells overexpressed HMGB1, whose molecular weight was identical to that of the native protein, whereas an additional higher-molecular-weight band corresponding to MIKSP-HMGB1 was detected in AdMIKSP-HMGB1-infected cells (Fig. 2A). Moreover, ELISA demonstrated that the culture medium from AdMIKSP-HMGB1-infected HeLa cells contained markedly higher HMGB1 levels than the media from AdHMGB1-, AdGFP-infected, or uninfected control cells did (Fig. 2B). Furthermore, serum HMGB1 concentrations were significantly elevated in AdMIKSP-HMGB1-infected mice (Fig. 2C). These results confirmed that AdMIKSP-HMGB1 effectively mediated the secretory expression of HMGB1.

Next, we examined the effects of adenovirus-mediated HMGB1 secretion on liver inflammation. qRT–PCR analysis revealed that AdMIKSP-HMGB1 infection upregulated the expression of proinflammatory cytokines such as tumor necrosis factor-alpha (TNF-α), interleukin (IL)-6, IL-17 A, and monocyte chemotactic protein-1 (MCP-1) in mouse livers (Supplementary Fig. 1A–D). Elevated hepatic expression of CD45, a common leukocyte antigen, in AdMIKSP-HMGB1-infected mice indicated that extracellular HMGB1 induced inflammatory cell infiltration (Supplementary Fig. 1E). Although serum ALT, AST, and LDH activities did not significantly differ among the four groups (Supplementary Fig. 1F-H), increased MPO activity and MDA levels, along with reduced SOD activity, in AdMIKSP-HMGB1-infected livers suggested that extracellular HMGB1 caused oxidative liver injury (Supplementary Fig. 1I-K).

With respect to liver fibrosis induced by secretory HMGB1, increased hydroxyproline content (Fig. 2D), increased collagen deposition (Fig. 2E), and elevated expression of collagen type I alpha 2 (Col1A2) and collagen type III alpha 1 (Col3A1) (Fig. 2F, G, J) were detected in the AdMIKSP-HMGB1-infected mice, compared with those in the other three groups, indicating that extracellular HMGB1 triggered fibrogenesis. Consistent with these findings, significantly upregulated expression levels of hepatic vimentin (Fig. 2H, J) and α-SMA (Fig. 2I, J) in AdMIKSP-HMGB1-infected mice indicated that HSC activation was induced by secretory HMGB1. Moreover, elevated expression levels of transforming growth factor-β1 (TGF-β1), platelet-derived growth factor (PDGF)-A, PDGF-B, connective tissue growth factor (CTGF), plasminogen activator inhibitor-1 (PAI-1), and TGF-βRII (the dominant receptor of TGF-βs) expression in AdMIKSP-HMGB1-infected mouse livers further supported the profibrotic role of secretory HMGB1 (Table 1).

**Table 1:** Adenovirus-mediated secretory expression of HMGB1 upregulates the profibrtoic cytokines and TGF-βRⅡ in mouse livers.

	NC (*n* = 6)	AdGFP (*n* = 6)	AdHMGB1 (*n* = 6)	AdMIKSP-HMGB1 (*n* = 6)
TGF-β1	1.01 ± 0.03	1.01 ± 0.05	0.97 ± 0.04	1.34 ± 0.20^††^
PDGF-A	1.00 ± 0.08	0.99 ± 0.11	1.06 ± 0.08	1.62 ± 0.23^††^
PDGF-B	0.98 ± 0.03	1.05 ± 0.16	1.10 ± 0.08	1.54 ± 0.24^††^
CTGF	1.01 ± 0.05	1.02 ± 0.04	1.08 ± 0.08	1.46 ± 0.19^††^
PAI-1	0.99 ± 0.03	1.13 ± 0.19	1.05 ± 0.10	2.38 ± 0.30^††^
TGF-βRⅡ	1.01 ± 0.08	0.89 ± 0.14	0.86 ± 0.26	1.35 ± 0.29^†^

Results are expressed as means ± SEM

^†^*P* < 0.05 and ^††^*P* < 0.01, vs. NC, AdGFP or AdHMGB1 group

### Immunization with PfTrx-HMGB1^17^(95–111) and HBcΔ-HMGB1^34^(150–183) Efficiently Elicits Neutralizing Antibodies against HMGB1 in Mice

**Fig. 3:**
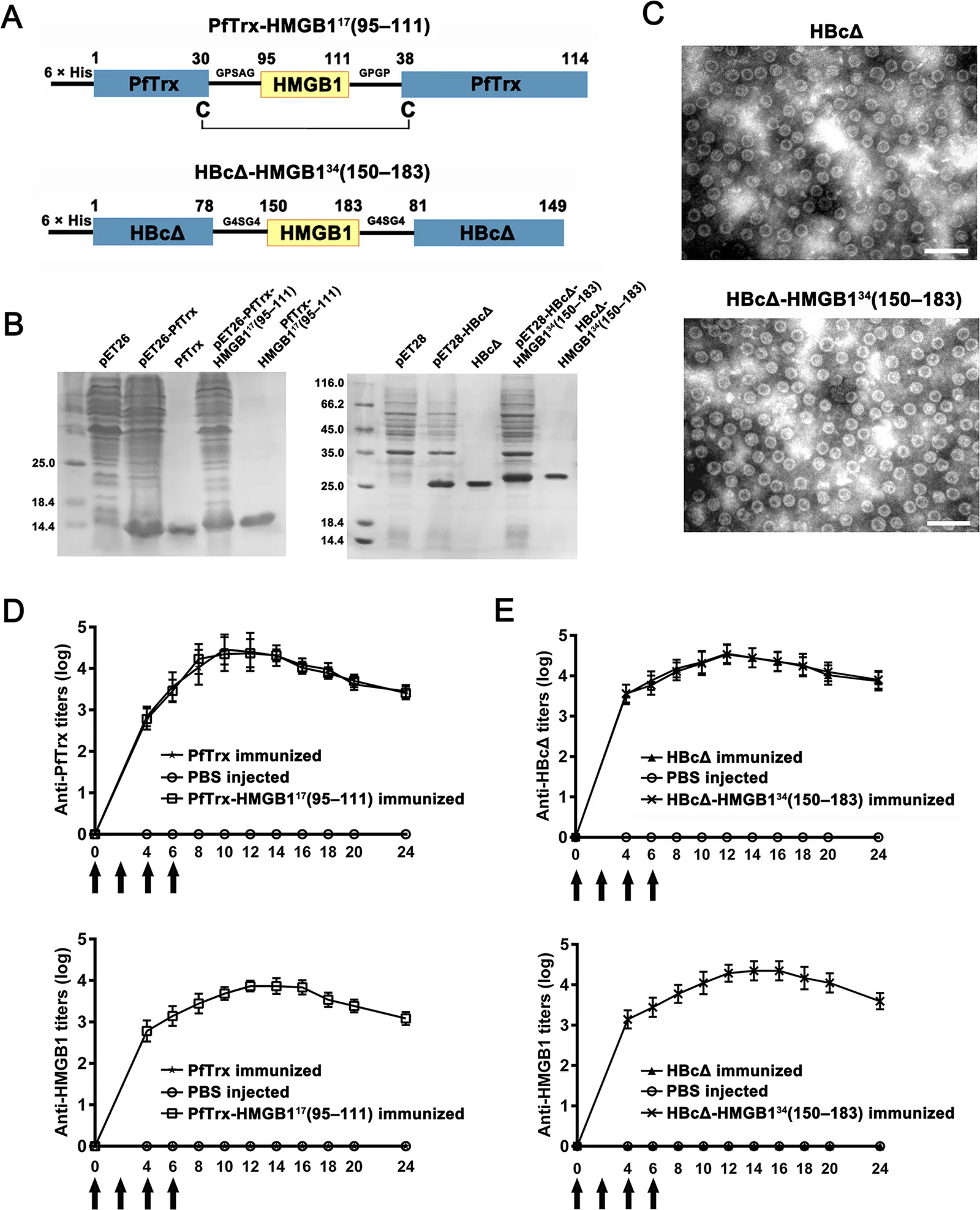
Preparation of the anti-HMGB1 vaccines and verification of their antigenecity. **A** Schematic diagrams of PfTrx-HMGB1^17^(95–111) and HBcΔ-HMGB1^34^(150–183). **B** SDS–PAGE followed by Commassie Brilliant Blue staining of the expressed and purified recombinant proteins. **C** Electron microscopy observation of HBcΔ and HBcΔ-HMGB1^34^(150–183) VLPs. Scale bars = 100 nm. (D–E) Mouse serum antibody titers after the immunizations. Arrows indicate the immunizations

Using computer-aided prediction, HMGB1^17^(95–111) and HMGB1^34^(150–183) were selected as B-cell epitopes because they encompass the regions through which HMGB1 binds to TLR4 and RAGE, respectively. We initially attempted to construct VLP vaccines by inserting these two fragments into the major immunodominant region (c/e1 epitope) of a C-terminus (aa 150–183)-truncated and MIR (aa 79–80)-deleted HBc (HBcΔ) [22]. However, the insertion of HMGB1^17^(95–111) impaired the self-assembly capacity of HBcΔ; therefore, PfTrx was chosen as the carrier for HMGB1^17^(95–111) (schematic diagrams shown in Fig. 3A). PfTrx, PfTrx-HMGB1^17^(95–111), HBcΔ, and HBcΔ-HMGB1^34^(150–183) were efficiently expressed in *Escherichia coli* as soluble proteins and were subsequently purified by using nickel nitrilotriacetic acid affinity chromatography (Fig. 3B). Following size-exclusion chromatography, electron microscopy revealed that the purified HBcΔ and HBcΔ-HMGB1^34^(150–183) preparations contained abundant spherical VLPs approximately 20 nm in diameter (Fig. 3C). Four consecutive immunizations (50 µg per mouse per dose), administered two weeks apart, with PfTrx-HMGB1^17^(95–111) and HBcΔ-HMGB1^34^(150–183) elicited HMGB1-specific antibodies, with the ELISA titers reaching 1:5,1200. The antibody levels persisted for 4–6 weeks before gradually declining (Fig. 3D, E).

**Fig. 4:**
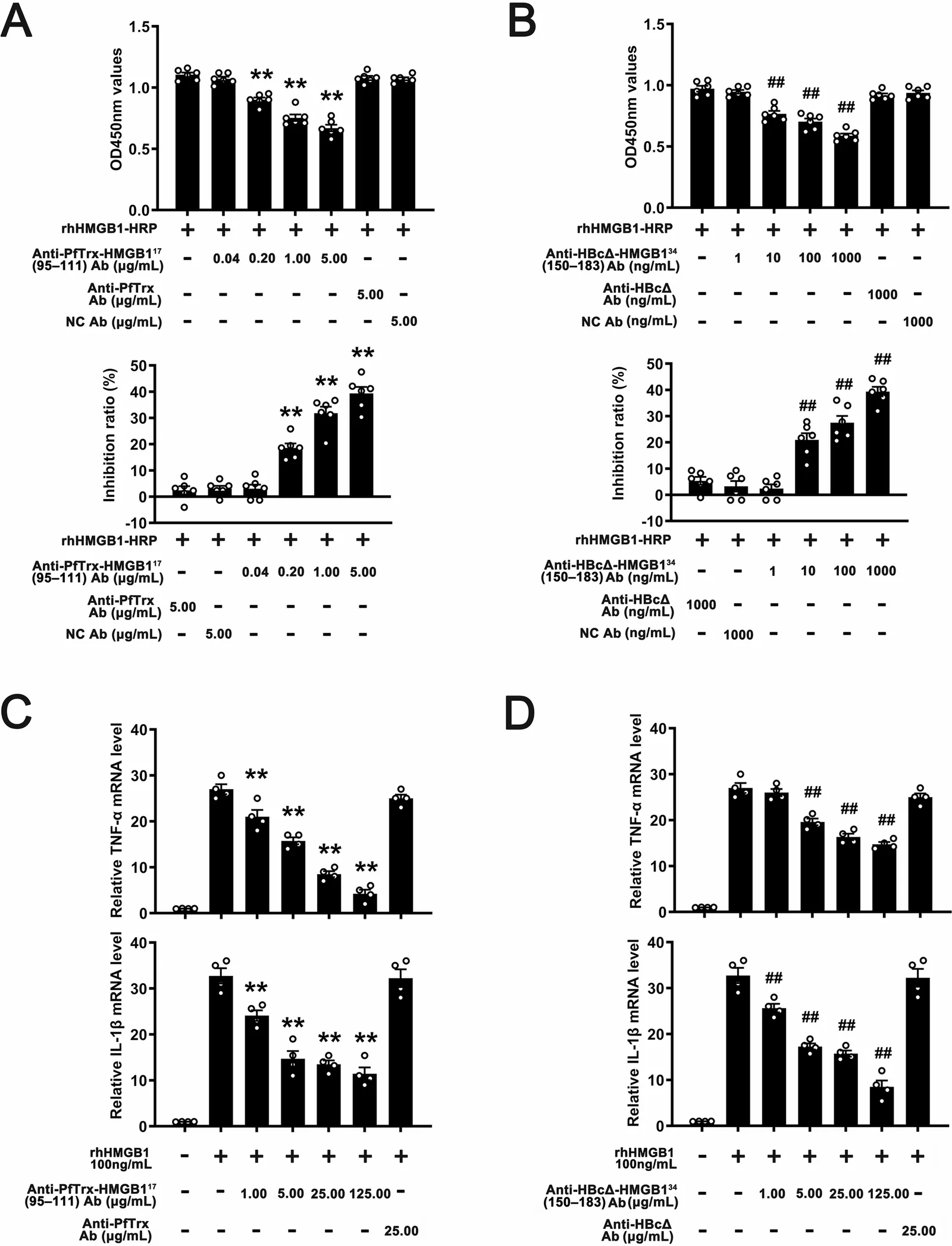
The antibodies block the binding of HMGB1 to TLR4 or RAGE and abolish HMGB1-induced TNF-α and IL-1β expression in THP-1 cells. **A**–**B** Competitive ELISA for evaluating the abilities of anti-PfTrx-HMGB1^17^(95–111) and anti-HBcΔ-HMGB1^34^(150–183) antibodies in blocking HMGB1 binding to TLR4 or RAGE. *n* = 6 independent experiments. ^**^P < 0.01 vs. rhHMGB1-HRP group, anti-PfTrx Ab group or NC Ab group. ^##^*P* < 0.01 vs. rhHMGB1-HRP group, Anti-HBcΔ Ab group or NC Ab group. **C**–**D** TNF-α and IL-1β mRNA expression in THP-1 cells. ^**^P < 0.01 vs. blank group, rhHMGB1 group or rhHMGB1 + anti-PfTrx Ab group. ^##^*P* < 0.01 vs. blank group, rhHMGB1 group or rhHMGB1 + anti-HBcΔ Ab group. *n* = 4 independent experiments. Data are shown as mean ± SEM

The ability of the antibodies to inhibit HMGB1 binding to TLR4 or RAGE was assessed via competitive ELISA. As shown in Fig. 4A, B, the OD at 450 nm (OD_450_) was negatively correlated with the concentrations of anti-PfTrx-HMGB1^17^(95–111) and anti-HBcΔ-HMGB1^34^(150–183) antibodies, confirming that these anti-HMGB1 antibodies effectively blocked the binding of HMGB1 to TLR4 and RAGE. The neutralizing effects of vaccine-elicited antibodies were further examined in THP-1 cells. qRT‒PCR analysis revealed that rhHMGB1 markedly upregulated TNF-α and IL-1β expression in THP-1 cells, whereas antibodies induced by immunization with either PfTrx-HMGB1^17^(95–111) or HBcΔ-HMGB1^34^(150–183) abolished these effects in a dose-dependent manner (Fig. 4C, D).

Notably, no behavioral abnormalities or apparent adverse effects were observed in the immunized mice up to euthanasia 24 weeks after the initiation of immunization. Histological examination of vital organs revealed no discernible lesions. To evaluate the potential impact of immunizations on antibacterial immunity, 50 mice were grouped and immunized as described above, and CLP was performed 1 week after the fourth immunization. The survival rates did not markedly differ among the five groups, indicating that the immunizations caused negligible impairment of antibacterial immunity (Supplementary Fig. 2).

### PfTrx-HMGB1^17^(95–111) and HBcΔ-HMGB1^34^(150–183) Immunizations Ameliorate CCl_4_-Induced Liver Injury and Inflammation

**Fig. 5:**
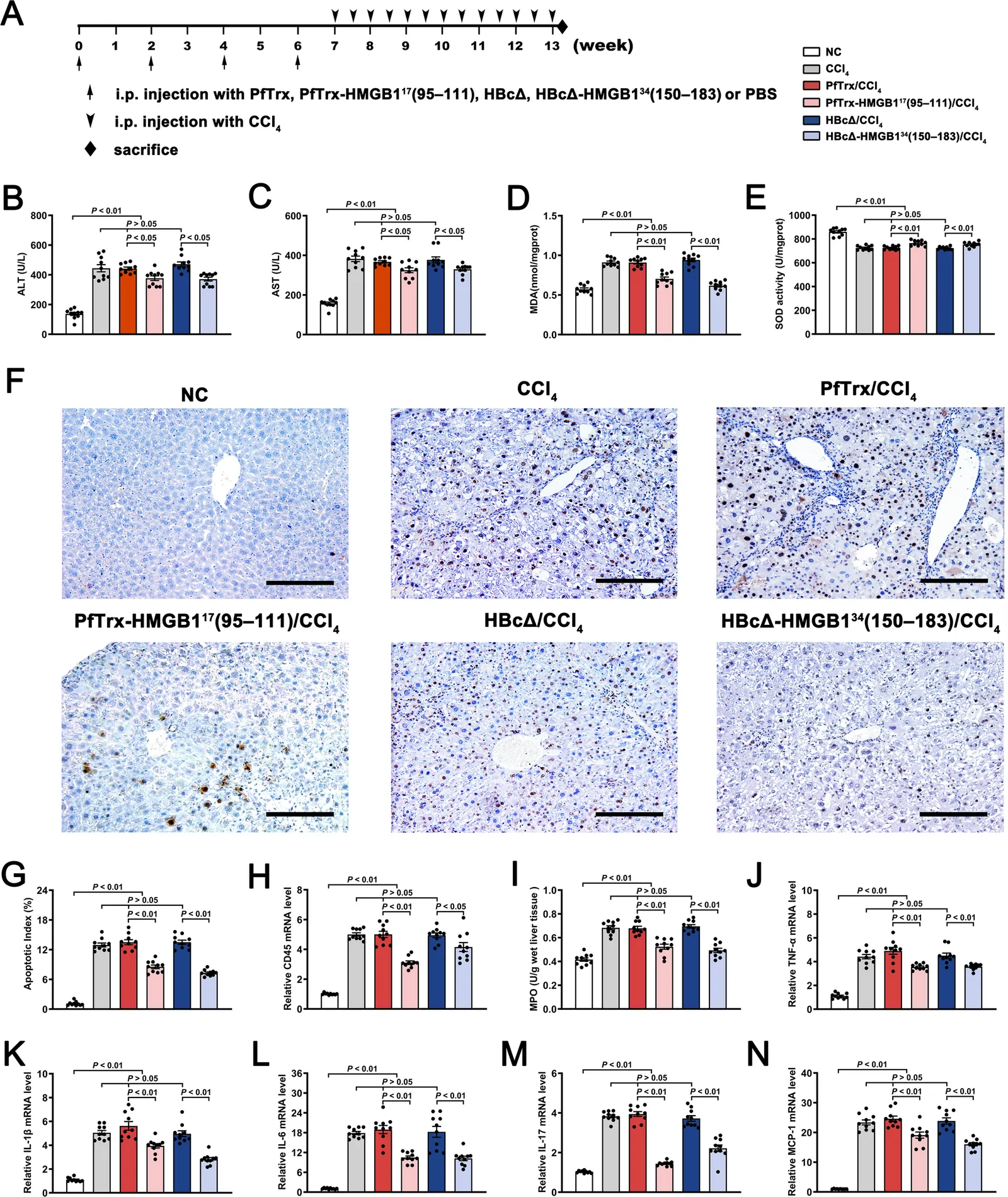
PfTrx-HMGB1^17^(95–111) and HBcΔ-HMGB1^34^(150–183) immunizations ameliorate CCl_4_-induced liver injury and inflammation. **A** Schematic illustration of the vaccination followed by modeling of hepatic fibrosis. **B**–**C** Mouse serum ALT and AST activities. **D**–**E** Mouse liver tissue MDA contents and SOD activities. **F** TUNEL assay for apoptotic cells in the mouse livers. **G** Apoptotic indices. **H** Hepatic CD45 mRNA levels. **I** MPO activities in liver tissues. **J**–**N** Hepatic TNF-α, IL-1β, IL-6, IL-17 A, and MCP-1 mRNA expression. *n* = 10 mice per group. Data are shown as mean ± SEM. Scale bars = 100 μm

Owing to the long immunization period (at least 8 weeks) and the relatively comparatively short duration required to induce liver fibrosis in mice, we performed immunization prior to modeling to assess the “preventive” efficacy of the vaccines against CCl_4_-induced liver fibrosis (the protocol is illustrated in Fig. 5A).

After six weeks of CCl_4_ intoxication, we first assessed the effects of vaccination on liver injury by measuring serum aminotransferase activities and hepatocyte apoptosis. Both PfTrx-HMGB1^17^(95–111) and HBcΔ-HMGB1^34^(150–183) immunizations attenuated the CCl_4_-induced increases in serum ALT and AST activities (Fig. 5B, C). The increase in the MDA content and the reduction in SOD activity in the liver tissues of CCl_4_-treated mice were also significantly mitigated by vaccination with either PfTrx-HMGB1^17^(95–111) or HBcΔ-HMGB1^34^(150–183), indicating that HMGB1 neutralization protected against CCl_4_-induced oxidative damage (Fig. 5D, E). TUNEL assay demonstrated that the AI values in both the PfTrx-HMGB1^17^(95–111)/CCl_4_ and HBcΔ-HMGB1^34^(150–183)/CCl_4_ groups were significantly lower than those in any of the other three CCl_4_-treated groups (Fig. 5F, G). Cleaved caspase-3 IHC also confirmed that hepatocyte apoptosis was decreased in these HMGB1 vaccine groups (Supplementary Fig. 3). The lower hepatic CD45 mRNA expression levels (Fig. 5H) and tissue MPO activities (Fig. 5I) and the decreased expression levels of proinflammatory cytokines such as TNF-α, IL-1β, IL-6, IL-17A, and MCP-1 in HMGB1 vaccine-immunized mice than in CCl_4_-treated mice (Fig. 5J-N), indicated that blocking the HMGB1–TLR4 and HMGB1–RAGE pathways had anti-inflammatory effects.

The neutralizing effects of the antibodies (1 µg/mL) on HMGB1 (100 ng/mL)-induced proinflammatory cytokine production were examined in LX-2 and RAW 264.7 cells. rhHMGB1 induced TNF-α, IL-1β, IL-6, and MCP-1 expression in both cell types, whereas antibodies from PfTrx-HMGB1^17^(95–111)- and HBcΔ-HMGB1^34^(150–183)-immunized mice abrogated these effects (Supplementary Fig. 4).

### Vaccination Against HMGB1 Alleviates CCl_4_-Induced Hepatic Fibrosis in Mice

**Fig. 6:**
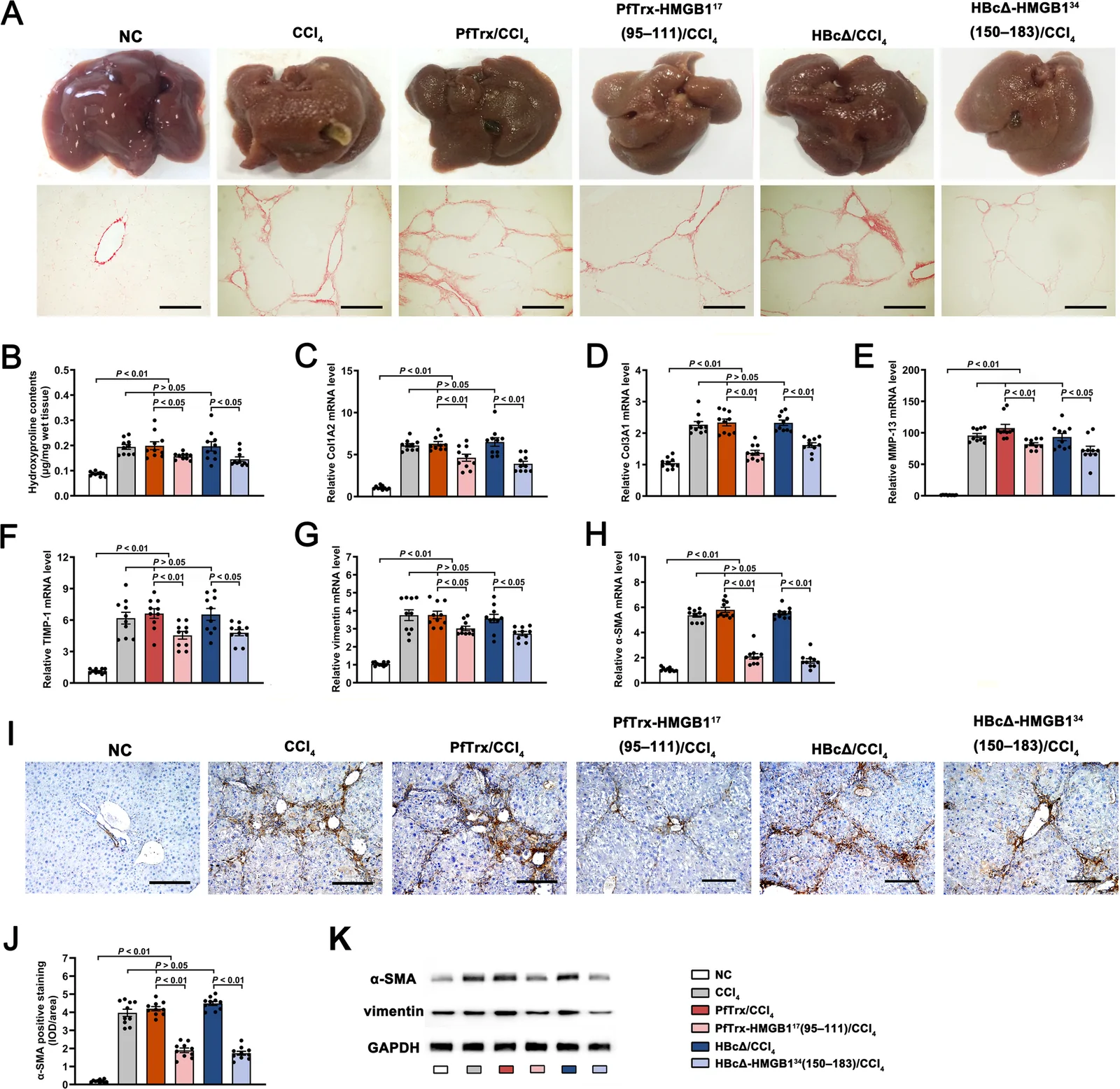
Vaccination against HMGB1 attenuates CCl_4_-induced hepatic fibrosis in mice. **A** Macroscopic appearances of the mouse livers and Sirius Red staining for the liver sections after six weeks of CCl_4_ treatment. Scale bars = 200 μm. **B** Hepatic hydroxyproline contents. **C**–**H** Col1A2, Col3A1, MMP-13, TIMP-1, vimentin and α-SMA mRNA expression. **I** IHC for α-SMA. Scale bars = 100 μm. **J** α-SMA positive staining analysis. **K** Western blotting analysis of the hepatic α-SMA and vimentin. *n* = 10 mice per group. Data are shown as mean ± SEM

**Fig. 7:**
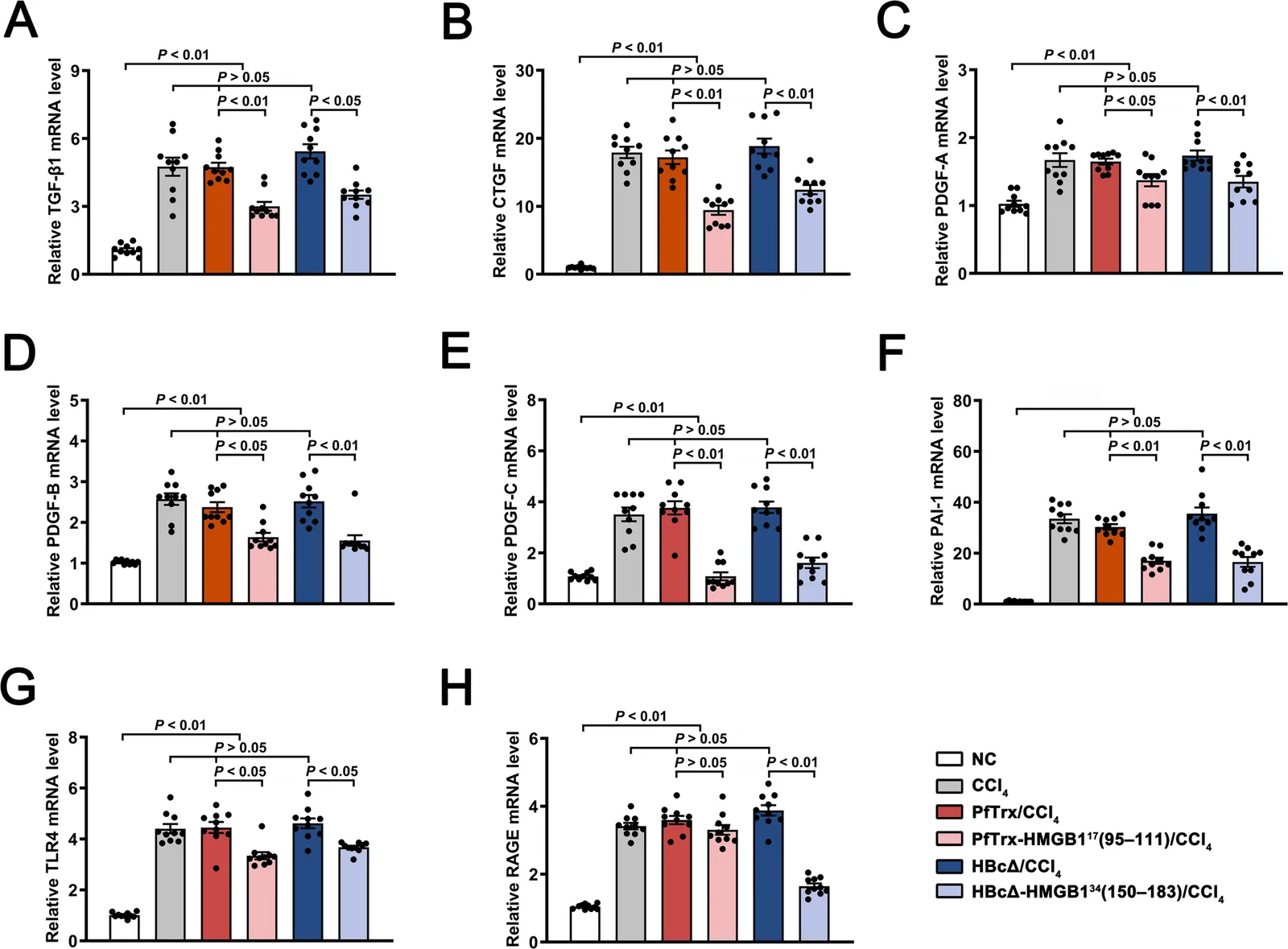
The vaccinations mitigates the upregulation of profibrotic cytokines and HMGB1 receptors in the fibrotic mouse livers. **A** TGF-β1, (**B**) CTGF, (**C**) PDGF-A, (**D**) PDGF-B, (**E**) PDGF-C, (**F**) PAI-1, (**G**) TLR4, and (**H**) RGAE mRNA levels in the mouse livers. *n* = 10 mice per group. Data are shown as mean ± SEM

After six weeks of CCl_4_ intoxication, the macroscopic appearance of the livers, Sirius Red staining of liver sections (Fig. 6A), and hepatic hydroxyproline content (Fig. 6B) revealed that vaccination with either PfTrx-HMGB1^17^(95–111) or HBcΔ-HMGB1^34^(150–183) significantly attenuated hepatic fibrosis in mice. Consistent with the reduced extracellular matrix (ECM) deposition, hepatic Col1A2 (Fig. 6C), Col3A1 (Fig. 6D), matrix metalloproteinase (MMP)-13 (Fig. 6E), and tissue inhibitor of metalloproteinase-1 (TIMP-1) (Fig. 6F) mRNA expression in the PfTrx-HMGB1^17^(95–111)/CCl_4_ and HBcΔ-HMGB1^34^(150–183)/CCl_4_ groups were significantly lower than those in either their respective carrier protein-immunized groups or the CCl_4_-only group. HSC activation represents a pivotal event in the progression of liver fibrosis [3, 4]. IHC, qRT‒PCR, and western blotting revealed that PfTrx-HMGB1^17^(95–111) and HBcΔ-HMGB1^34^(150–183) immunizations markedly mitigated the overexpression of the myofibroblast markers α-SMA and vimentin in fibrotic mouse livers (Fig. 6G-K), demonstrating the inhibitory effects of these immunizations on HSC activation. Profibrotic cytokines such as TGF-β1, PDGFs, CTGF, and PAI-1 are among the most potent drivers of HSC activation and liver fibrosis [4]. As shown in Fig. 7, the overexpression of TGF-β1, CTGF, PDGF-A, PDGF-B, PDGF-C, and PAI-1 in CCl_4_-intoxicated mouse livers was significantly attenuated by preimmunization with PfTrx–HMGB1^17^(95–111) and HBcΔ-HMGB1^34^(150–183) (Fig. 7A–F).

In addition, the mRNA expression of both TLR4 and RAGE was substantially upregulated in mouse fibrotic livers. PfTrx-HMGB1^17^(95–111) and HBcΔ-HMGB1^34^(150–183) immunizations significantly mitigated TLR4 overexpression. RAGE upregulation was markedly alleviated by HBcΔ-HMGB1^34^(150–183) but only marginally attenuated by PfTrx-HMGB1^17^(95–111) in CCl_4_-intoxicated mouse liver tissues (Fig. 7G, H).

### Anti-HMGB1 Antibodies Abolish HMGB1-Induced HSC Activation and Profibrotic Cytokine Production in Liver Cells

**Fig. 8:**
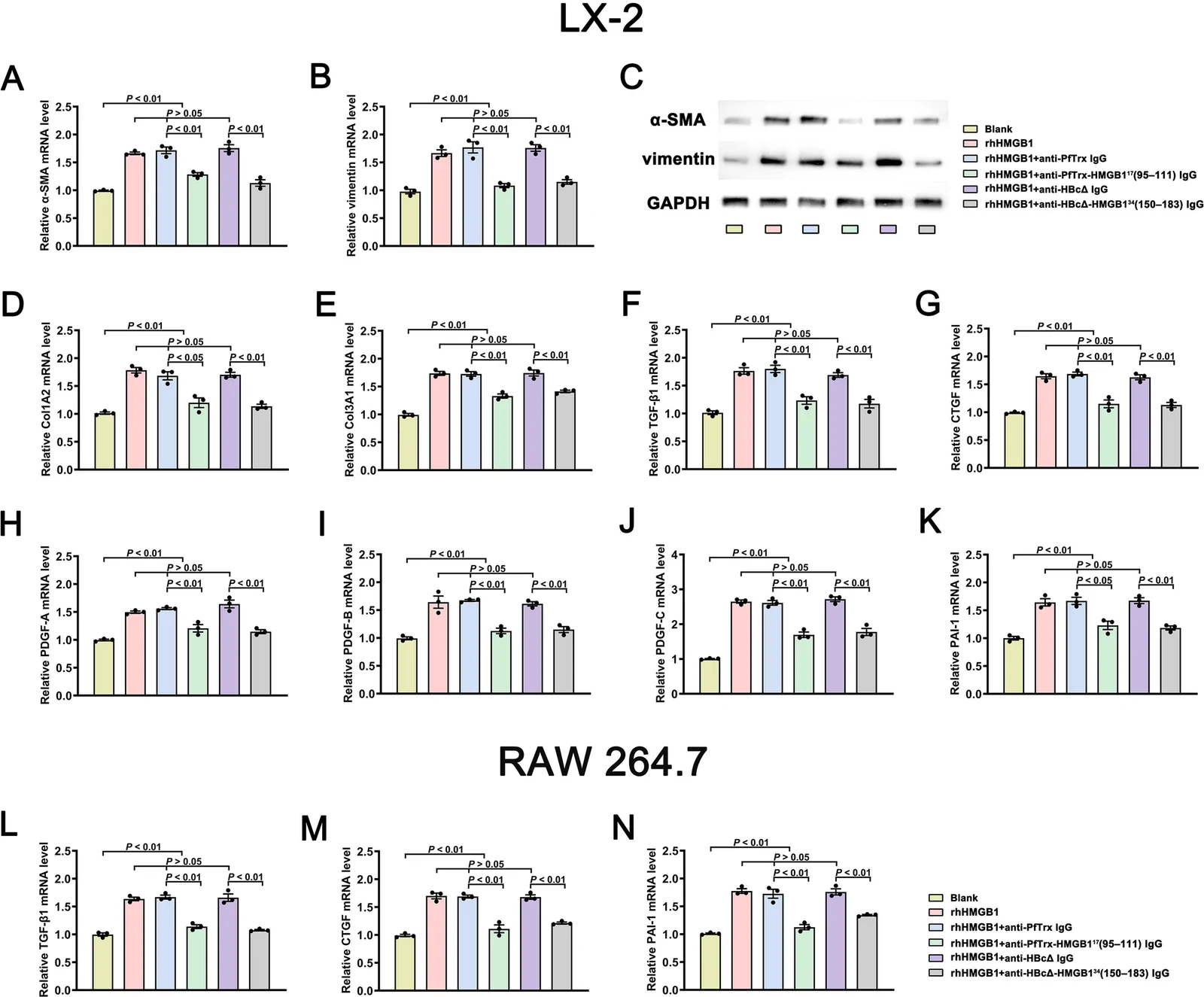
Anti-HMGB1 antibodies abolish HMGB1-induced HSC activation and profibrotic cytokine production in LX-2 and RAW 264.7 cells. **A**–**B** α-SMA and vimentin mRNA levels in LX-2 cells. **C** Western blotting analysis for α-SMA and vimentin. **D**–**K** Col1A2, Col3A1, TGF-β1, CTGF, PDGF-A, PDGF-B, PDGF-C, and PAI-1 mRNA levels in LX-2 cells. (L–N) TGF-β1, CTGF, and PAI-1 mRNA expression in RAW 264.7 cells. *n* = 3 independent experiments. Data are shown as mean ± SEM

rhHMGB1 upregulated the expression of α-SMA, vimentin, Col1A2, and Col3A1 in LX-2 cells, whereas antibodies against PfTrx-HMGB1^17^(95–111) or HBcΔ-HMGB1^34^(150–183) abrogated these effects (Fig. 8A–E). These results indicate that the profibrotic effect of HMGB1 is attributable, at least in part, to its interactions with TLR4 and RAGE in HSCs and the subsequent activation of HSCs. Moreover, the antibodies also abrogated the rhHMGB1-induced expression of TGF-β1, CTGF, PDGF-A, PDGF-B, PDGF-C and PAI-1 in LX-2 cells (Fig. 8F–K) and the expression of TGF-β1, CTGF and PAI-1 in cultured RAW 264.7 cells (Fig. 8L–N).

We additionally tested the neutralizing effects of the antibodies against disulfide HMGB1 in LX-2 and RAW 264.7 cells. Like rhHMGB1, disulfide HMGB1 upregulated the expression of α-SMA, vimentin, Col1A2, Col3A1, MMP-13, TIMP-1, TGF-β1, CTGF, PAI-1, TNF-α, IL-1β, IL-6, and MCP-1 in LX-2 cells, whereas antibodies against PfTrx-HMGB1^17^(95–111) or HBcΔ-HMGB1^34^(150–183) abrogated these effects (Supplementary Fig. 5A–M). Additionally, these antibodies attenuated disulfide HMGB1-induced overexpression of MMP-13, TIMP-1, CTGF, TGF-β1, PAI-1, TNF-α, IL-1β, IL-6, and MCP-1 in RAW 264.7 cells (Supplementary Fig. 5N–V).

## Discussion

Previous studies have suggested that HMGB1 plays a profibrotic role in various organs and tissues, including the lungs, kidneys, liver, and heart [26]. However, most in vivo studies have focused on HMGB1 knockout, knockdown, overexpression, neutralization, or exogenous administration in fibrotic models. Therefore, a critical question remains as to whether HMGB1 can independently initiate and sustain fibrosis. Moreover, genetic manipulations such as HMGB1 knockout, knockdown, or overexpression primarily affect nuclear HMGB1, which complicates interpretation because intracellular HMGB1 exerts protective functions, in contrast to the detrimental effects of extracellular HMGB1. Although the results of in vitro experiments also revealed that extracellular HMGB1 promotes fibroblast and myofibroblast accumulation, ECM production, and profibrotic cytokine expression [9, 27], the results of the present study confirm mainly that HMGB1 perpetuates established fibrotic processes rather than initiating them. In this study, by using recombinant adenovirus to mediate the secretion of HMGB1, we confirmed that extracellular HMGB1 is sufficient to initiate and sustain both inflammation and fibrogenesis in the liver. Notably, the observation that pulmonary inflammatory cell infiltration and fibrogenesis occurred in the same group of mice reinforces the pivotal role of extracellular HMGB1 in inflammation and fibrosis (our unpublished data).

Anti-cytokine vaccines offer promising clinical potential as alternative “next-generation” therapeutic approaches to antibody-based therapies. For instance, the IL-1β vaccine has been applied in the treatment of type 2 diabetes and arthritis [28, 29], the TNF-α vaccine in arthritis [30, 31], and the EGF vaccine in the clinical management of non-small cell lung cancer [32]. Since 2008, our research group has been committed to developing anticytokine vaccines and has successively developed several anticytokine vaccines targeting PDGF-B [33], TGF-β1 [34], CTGF [22], and IL-1β [25]; moreover, several anticytokine vaccines for fibrosis have been developed and tested in animal models of liver or lung fibrosis. Unlike chemical compounds, which may bind a wide range of molecules, vaccines can elicit antibodies that precisely target specific pathogenic cytokines or growth factors, thereby avoiding potential adverse effects. Although therapeutic antibodies can specifically neutralize the pathogenic effects of their antigens, repeated injections of these expensive reagents limit their clinical application. In contrast, vaccines offer prominent advantages, such as their specificity, low cost, and long-lasting effects, making them promising therapeutic strategies for the future. In this study, we designed and constructed PfTrx-HMGB1^17^(95–111) and HBcΔ-HMGB1^34^(150–183), which carry polypeptide fragments responsible for HMGB1-mediated activation of TLR4 and RAGE, respectively [1, 35]. Based on well-established protocols in the literature [36] and our prior experimental experience [22, 25, 33, 34], we evaluated the efficacy and safety of two doses (50 µg and 100 µg) of the recombinant proteins and ultimately determined that a 50 µg dose successfully elicited robust immune responses without causing adverse effects. Antibodies targeting the two HMGB1 epitopes can block the proinflammatory and profibrotic actions of the HMGB1 B-box while preserving the antagonistic effect of the A-box. Our prior experiments corroborated this hypothesis: vaccines prepared by coupling HMGB1 A-box-derived antigenic peptides (aa 53–63 and aa 67–78) with KLH exacerbated rather than protected against CCl_4_-induced liver fibrosis. Our results demonstrating that immunization with PfTrx-HMGB1^17^(95–111) or HBcΔ-HMGB1^34^(150–183) protects mice from CCl_4_-induced liver injury, inflammation, and fibrosis suggest that anti-HMGB1 vaccines may be developed as promising therapeutic strategies for liver fibrosis.

Controversies exist regarding to the roles of TLR4 and RAGE in inflammation and fibrosis. Although the proinflammatory effects of TLR4 and the proinflammatory and profibrotic functions of RAGE in the liver are widely recognized, inconsistent results have been reported regarding the role of TLR4 in liver fibrosis. Ge et al. silenced RAGE or TLR4 in HSCs and found that HMGB1 upregulated type I collagen in HSCs via RAGE rather than TLR4 [6]. RAGE ablation or RAGE neutralization, but not TLR4 ablation, in HSCs prevented CCl_4_-induced liver fibrosis in mice [6], suggesting that HMGB1 signaling via RAGE, rather than TLR4, mediates its profibrogenic effects. Conversely, Seki et al. reported that TLR4-mutant mice (C3H/HeJ) displayed less fibrosis in CCl_4_-, thioacetamide-, or BDL-induced fibrosis models [37], indicating that TLR4 is also involved in liver fibrosis. TLR4 sustains HSC survival by upregulating antiapoptotic mediators such as baculovirus IAP repeat-containing protein 2 (Birc2) and Birc3. In HSCs, TLR4 activates Kupffer cells and downregulates the expression of the TGF-β pseudoreceptor Bambi, thereby sensitizing HSCs to TGF-β-induced signaling [37]. Additionally, TLR4 promotes hepatic fibrogenesis by activating proinflammatory pathways in activated HSCs. Paik et al. reported that stimulation of cultured HSCs with lipopolysaccharide activates nuclear factor kappa B (NF-κB) and JNK and upregulates chemokines and adhesion molecules via TLR4 [38]. Our results demonstrate that both inflammation and fibrosis in mouse livers are attenuated by immunization with either PfTrx-HMGB1^17^(95–111) or HBcΔ-HMGB1^34^(150–183) and that blocking either the HMGB1–TLR4 pathway or the HMGB1–RAGE pathway significantly inhibits HMGB1-induced HSC activation, as well as proinflammatory and profibrotic cytokine expression. These findings indicate that the HMGB1–TLR4 and HMGB1–RAGE pathways similarly mediate inflammation and fibrosis in the liver. Moreover, TLR4 and RAGE may act synergistically in these processes. Our results show that blocking the HMGB1–RAGE pathway reduces TLR4 expression, while blocking the HMGB1–TLR4 pathway decreases RAGE expression in fibrotic mouse livers. Consistent with our findings, Zhong et al. reported that knocking out RAGE decreases TLR4 expression and vice versa in peritoneal macrophages from pseudofracture mice [39]. Notably, compared with the PfTrx-HMGB1^17^(95–111), the HBcΔ-HMGB1^34^(150–183) vaccine more strongly inhibited RAGE signaling in vivo. This observation can likely be attributed to the pleiotropic characteristics of HMGB1 and the intricacy of its signaling network. HMGB1 induces liver fibrosis via multiple receptors, since the two vaccines target different epitopes of HMGB1, they may preferentially interfere with different receptor-binding interfaces. Although the PfTrx-HMGB1^17^(95–111) vaccine exhibited relatively weaker inhibition of RAGE, its protective efficacy remained intact. This implies that this vaccine construct may exert antifibrotic effects by more effectively blocking other crucial pathways, like the HMGB1–TLR4 axis, or by broadly neutralizing extracellular HMGB1 activity.

Notably, the HMGB1–RAGE pathway may exert distinct effects in different organs. In contrast to immunization with PfTrx-HMGB1^17^(95–111), immunization with HBcΔ-HMGB1^34^(150–183) exacerbated bleomycin-induced pulmonary inflammation and fibrosis, although antibodies produced by either vaccine attenuated HMGB1-induced fibroblast activation and proinflammatory and profibrotic cytokine production in cultured cells. Moreover, vaccination with PfTrx-HMGB1^17^(95–111) attenuated the downregulation of RAGE expression, whereas HBcΔ-HMGB1^34^(150–183) immunization further upregulated TLR4 expression following bleomycin treatment (our unpublished data). Further studies are warranted to elucidate these discrepancies. Nevertheless, our findings suggest that vaccines targeting specific domains can also serve as valuable tools for investigating the functions and underlying mechanisms of extracellular proteins.

However, several limitations should be acknowledged. First, in this study, the efficacy of the HMGB1 vaccines was validated in CCl_4_-induced mouse models, which may not fully recapitulate the complexity of human liver fibrosis etiologies such as NASH or viral hepatitis. Second, while we highlighted the blockade of the TLR4 and RAGE pathways, HMGB1 also interacts with other receptors (e.g., CXCR4 and TLR9); whether blocking these specific epitopes triggers compensatory signaling warrants further investigation. Third, the biological activity of HMGB1 is highly dependent on the redox state of its cysteine residues (for instance, disulfide-bonded HMGB1 has chemotactic activity, while whereas fully reduced HMGB1 has cytokine-inducing activity). Whether the antibodies produced by the vaccine are equally effective at recognizing and neutralizing HMGB1 in different redox states still needs further confirmation. Finally, we performed immunization prior to modeling to assess the preventive efficacy of the vaccines against liver fibrosis. Future studies should assess the durability of the vaccine-induced humoral response and its ability to reverse established, late-stage fibrosis.

## Conclusions

Our research demonstrated that, in addition to its proinflammatory effects, extracellular HMGB1 can directly trigger liver fibrosis in mice. The HMGB1 vaccines PfTrx-HMGB1^17^(95–111) and HBcΔ-HMGB1^34^(150–183) specifically inhibited HMGB1 binding to TLR4 and RAGE, thereby effectively attenuating liver fibrosis both in vitro and in vivo experiments. These findings underscore the pivotal role of the HMGB1–TLR4/RAGE axis in liver inflammation and fibrosis, highlighting that vaccination-mediated blockade of this interaction has significant therapeutic potential for hepatic fibrosis.

## Supplementary Information

Supplementary Material 1.

## Data Availability

No datasets were generated or analysed during the current study.

## Abbreviations

Ab: Antibody
aa: Amino acids
AI: Apoptotic index
ALT: Alanine aminotransferase
ANOVA: One-way analysis of variance
ASH: Alcohol-associated steatohepatitis
α-SMA: Alpha-smooth muscle actin
AST: Aspartate aminotransferase
BDL: Bile duct ligation
Birc2: Baculovirus IAP repeat-containing protein 2
CCl_4_: Carbon tetrachloride
CD45: Leukocyte common antigen
CLP: Cecal ligation and puncture
Col1A2: Collagen type I alpha 2
Col3A1: Collagen type III alpha 1
CTGF: Connective tissue growth factor
DMEM: Dulbecco’s modified Eagle medium
ECM: Extracellular matrix
ELISA: Enzyme-linked immunosorbent assay
FBS: Fetal bovine serum
GFP: Green fluorescent protein
HBc: Hepatitis B core antigen
HMGB1: High mobility group box 1
HRP: Horseradish peroxidase
HSCs: Hepatic stellate cells
IHC: Immunohistochemistry
IL: Interleukin
i.p.: Intraperitoneal
JNK: Jun N-terminal kinase
KLH: Keyhole limpet hemocyanin
LDH: Lactate dehydrogenase
MCP-1: Monocyte chemotactic protein-1
MDA: Malondialdehyde
MIKSP: Murine immunoglobulin kappa signal peptide
MMP: Matrix metalloproteinase
MOI: Multiplicity of infection
MPO: Myeloperoxidase
NASH: Nonalcoholic steatohepatitis
NF-κB: Nuclear factor kappa B
PAI-1: Plasminogen activator inhibitor-1
PBS: Phosphate-buffered saline
PCNA: Proliferating cell nuclear antigen
PDGF: Platelet-derived growth factor
PfTrx: Pyrococcus furiosus thioredoxin
PFU: Plaque-forming unit
qRT-PCR: Quantitative real-time polymerase chain reaction
RAGE: Receptor for advanced glycation end products
rhHMGB1: Recombinant human HMGB1
SDS‒PAGE: Sodium dodecyl sulfate‒polyacrylamide gel electrophoresis
SEM: Standard error of the mean
shRNA: Short hairpin RNA
siRNA: Small interfering RNA
SOD: Superoxide dismutase
TGF-β1: Transforming growth factor-β1
TIMP-1: Tissue inhibitor of metalloproteinase-1
TLR4: Toll-like receptor 4
TNF-α: Tumor necrosis factor alpha
TUNEL: Terminal deoxynucleotidyl transferase dUTP nick end labeling
VLPs: Virus-like particles
