# Supplementary material for: Vaccination Against HMGB1 Protects Mice from CCl4-Induced Liver Injury, Inflammation, and Fibrosis
Source: Inflammation. 2026 Aug 1;49(1):208. doi: 10.1007/s10753-026-02578-4 (PMC13719786; doi:10.1007/s10753-026-02578-4)
Supplement: Supplementary file 1 [file 10753_2026_2578_MOESM1_ESM.docx]

Supplementary Figure 1 Supplementary Fig 1. Extracellular HMGB1 provokes inflammation and oxidative injury in mouse livers. (A–E) Hepatic TNF-α, IL-6, IL-17A, MCP-1, and CD45 mRNA expression in the mice. (F–H) Mouse serum ALT, AST, and LDH activities. (I–K) Mouse liver tissue MPO activity, MDA content, and SOD activity. n = 6 mice per group.  Data are shown as mean ± SEM.
Supplementary Figure 2 Supplementary Fig 2. The immunized mice showed no obvious organ injury or impairment to anti-infection immunity. (A) HE staining for the heart, lung, liver, kidney of the mice underwent four-round immunization followed by eighteen-week maintenance. Scale bars = 100 μm. (B) Survival curve for the mice following cecal ligation and puncture.
Supplementary Figure 3 Supplementary Fig 3. HMGB1 vaccines ameliorate CCl4-induced liver injury and inflammation. (A) Cleaved caspase 3/P17/P19 staining for apoptotic cells in the mouse livers. (B) Cleaved caspase 3 positive staining analysis. Data are shown as mean ± SEM. Scale bars = 100 μm.
Supplementary Figure 3 Supplementary Fig 4. Anti-HMGB1 antibodies abolish rhHMGB1-induced pro-inflammatory cytokine expression in LX-2 and RAW 264.7 cells. (A–D) TNF-α, IL-1β, IL-6, and MCP-1 mRNA expression in LX-2 cells. (E–H) TNF-α, IL-1β, IL-6, and MCP-1 mRNA expression in RAW 264.7 cells. n = 3 independent experiments.  Data are shown as mean ± SEM.
Supplementary Figure 4 Supplementary Fig 5. Anti-HMGB1 antibodies neutralize disulfide HMGB1-induced HSC activation and proinflammatory/profibrotic cytokine expression in vitro. LX-2 cells or RAW 264.7 cells were treated with disulfide HMGB1 and/or anti-HMGB1 antibodies elicited by the vaccinations for 24h. (A–M) α-SMA, vimentin, Col1A2, Col3A1, MMP-13, TIMP-1, TGF-β1, CTGF, PAI-1, TNF-α, IL-1β, IL-6, and MCP-1 mRNA expression in LX-2 cells  (N–V) MMP-13, TIMP-1, CTGF, TGF-β1, PAI-1, TNF-α, IL-1β, IL-6, and MCP-1 mRNA expression in RAW 264.7 cells. n = 3 independent experiments.  Data are shown as mean ± SEM.
Supplementary Figure 6 Supplementary Fig 6. Raw uncropped images of western blotting.

Supplementary Table 1: Primers for qRT-PCR
Gene
species
Sequence
β-actin
mouse
5´- CCTCTATGCCAACACAGTGC -3´
5´- CACACAGAGTACTTGCGCTC -3´
human
5´- GAAGTGTGACGTGGACATCC -3´
5´- GATCCACACGGAGTACTTGC -3´
α-SMA
mouse
5´- GTACCACCATGTACCCAGGC -3´
5´- GCTGGAAGGTAGACAGCG -3´
human
5´- CTGGCATTGCCGACCGAATG -3´
5´- GATCCACATCTGCTGGAAGG -3´
CoL1A2
mouse
5´- CAGCTTGAACAAGGCTGTGC -3´
5´- CAATGTCAAGGAACGGCAGG -3´
human
5´- GGCTGTCATTCTACAGGGCTC -3´
5´- CAATATCAAGGAAGGGCAGGC -3´
CoL3A1

mouse
5´- CACAGTTCTAGAGGATGGCTG -3´
5´- CACACCAAATTCTTGATCAGGAC -3´
human
5´- CACCTACACAGTTCTGGAGG -3´
5´- CCACCAATGTCATAGGGTGC -3´
vimentin
mouse
5´- ACTAACCTGGAGTCACTTCC -3´
5´- TTGCTGGTACTGCACTGTTG -3´
human
5´- GGACACTTCTGATTAAGACGG -3´
5´- GCAGAAAGGCACTTGAAAGC -3´
TGF-β1
mouse
5´- CATGCCAACTTCTGTCTGGGA -3´
5´- CTTGCGACCCACGTAGTAGAC -3´
human
5´- GCTACCATGCCAACTTCTGC -3´
5´- CGATCATGTTGGACAGCTGC -3´
PDGF-A
mouse
5´- AACACCAGCAGCGTCAAGTG -3´
5´- TTCAGGTTGGAGGTCGCAC -3´
human
5´- AACACGAGCAGTGTCAAGTG -3´
5´- TCCCGATAATCCGGATTCAG -3´
PDGF-B
mouse
5´- CTCTCGGAACCTCATCGATC -3´
5´- GGCTTCTTTCGCACAATCTC -3´
human
5´- TGTGGCCAGCACACCAAGT -3´
5´- AGGTCACCATCTACAGCCACCTC -3´
PDGF-C
mouse
5´- TGGACTTGGACAGCCTCTAC -3´
5´- TGTTCCAGAGCCACATCAG -3´
human
5´- CCAGAAGATGACATATGCAAG -3´
5´- TAAGTCCAACTGCCATCTCTC -3´
CTGF
mouse
5´- ACCCGAGTTACCAATGACAATACC -3´
5´- CCGCAGAACTTAGCCCTGTATG -3´
human
5´- GTTACCAATGACAACGCCTC -3´
5´- GTATGTCTTCATGCTGGTGC -3´
PAI-1
mouse
5´- AGGATCGAGGTAAACGAGAG -3´
5´- GATCGGTCTATAACCATCTC -3´
human
5´- ACCCTCAGCATGTTCATTGC -3´
5´- TTCCTGAGGTCGACTTCAGT -3´
TNF-α
mouse
5´- GGAACTGGCAGAAGAGGCAC -3´
5´- GAGGGAGGCCATTTGGGAAC -3´
human
5´- GATCATCTTCTCGAACCCCG -3´
5´- CCTGGGAGTAGATGAGGTAC -3´
IL-1β
mouse
5´- CAAAATACCTGTGGCCTTGG -3´
5´- GGAACTCTGCAGACTCAAAC -3´
human
5´- TGTACCTGTCCTGCGTGTTG -3´
5´- GAAGACGGGCATGTTTTCTG -3´
IL-6
mouse
5´- GATGGATGCTACCAAACTGG -3´
5´- CTCTCTGAAGGACTCTGGC -3´
human
5´- AGAGCTGTGCAGATGAGTAC -3´
5´- GTCATGTCCTGCAGCCACTG -3´
IL-17A
mouse
F: 5´- GCTCCAGAAGGCCCTCAGA-3´
R: 5´- AGCTTTCCCTCCGCATTGA-3´
CD45
mouse
F: 5´- TCAATCTCTTGGAAAGTGCAG-3´
R: 5´- TTCTTGACTTGTCCATTCTGG-3´
MCP-1
mouse
5´-GATCCCAATGAGTAGGCTGG -3´
5´- CTGGACCCATTCCTTCTTGG-3´
human
5´- GCTATAGAAGAATCACCAGC-3´
5´- CCATGGAATCCTGAACCCAC-3´
TIMP-1

mouse
5´- ACCTATAGTGCTGGCTGTGG -3´
5´- GTAGTCCTCAGAGCCCACGA -3´
human
5´- TGGGGACACCAGAAGTCAAC -3´
5´- AAGTATCCGCAGACACTCTC -3´
MMP13
mouse
5´- CTCAGCAGGTTGAGGCTGAG -3´
5´- TCAGAGCCCAGAATTTTCTCC -3´
human
5´- GGTGATAAAGTAGATGCTGTC -3´
5´- CGCACTTCTGGAAGTATTAC -3´
TGF-βR II
mouse
5´- GAGCATGAAAGACAGTGTGC -3´
5´- ACACTCTGTGCTGTGAGACG -3´
HMGB1

TLR4

RAGE
mouse

mouse

mouse
5´- GAGAACATCCTGGCCTGTCC-3´
5´- TTCCTTTAGCTCGATATGCAGC-3´
5´- CCTGATGACATTCCTTCTTC -3´
5´- GTGTAAGCCATGCCATGC -3´
5´- GTCAGAGCTGACAGTGATCC -3´
5´- CTCAACCAACAGCTGAATGC -3´
